# Neuroprotective Effects of Aucubin against Cerebral Ischemia and Ischemia Injury through the Inhibition of the TLR4/NF-κB Inflammatory Signaling Pathway in Gerbils

**DOI:** 10.3390/ijms25063461

**Published:** 2024-03-19

**Authors:** Joon Ha Park, Tae-Kyeong Lee, Dae Won Kim, Ji Hyeon Ahn, Myoung Cheol Shin, Jun Hwi Cho, Moo-Ho Won, Il Jun Kang

**Affiliations:** 1 Department of Anatomy, College of Korean Medicine, Dongguk University, Gyeongju 38066, Republic of Korea; 2 Department of Food Science and Nutrition, Hallym University, Chuncheon 24252, Republic of Korea; 3 Department of Biochemistry and Molecular Biology, Research Institute of Oral Sciences, College of Dentistry, Gangneung-Wonju National University, Gangneung 25457, Republic of Korea; 4 Department of Physical Therapy, College of Health Science, Youngsan University, Yangsan 50510, Republic of Korea; 5 Department of Emergency Medicine, Kangwon National University Hospital, School of Medicine, Kangwon National University, Chuncheon 24289, Republic of Korea

**Keywords:** gliosis, hippocampus, iridoid glycoside, ischemia and reperfusion, neuroinflammation, proinflammatory cytokines

## Abstract

Aucubin, an iridoid glycoside, possesses beneficial bioactivities in many diseases, but little is known about its neuroprotective effects and mechanisms in brain ischemia and reperfusion (IR) injury. This study evaluated whether aucubin exhibited neuroprotective effects against IR injury in the hippocampal CA1 region through anti-inflammatory activity in gerbils. Aucubin (10 mg/kg) was administered intraperitoneally once a day for one week prior to IR. Neuroprotective effects of aucubin were assessed by neuronal nuclei (NeuN) immunofluorescence and Floro-Jade C (FJC) histofluorescence. Microgliosis and astrogliosis were evaluated using immunohistochemistry with anti-ionized calcium binding adapter protein 1 (Iba1) and glial fibrillary acidic protein (GFAP). Protein levels of proinflammatory cytokines interleukin1 beta (IL1β) and tumor necrosis factor alpha (TNFα) were assayed using enzyme-linked immunosorbent assay and Western blot. Changes in toll-like receptor 4 (TLR4)/nuclear factor-κB (NF-κB) signaling pathway were assessed by measuring levels of TLR4, inhibitor of NF-κB alpha (IκBα), and NF-κB p65 using Western blot. Aucubin treatment protected pyramidal neurons from IR injury. IR-induced microgliosis and astrogliosis were suppressed by aucubin treatment. IR-induced increases in IL1β and TNFα levels were significantly alleviated by the treatment. IR-induced upregulation of TLR4 and downregulation of IκBα were significantly prevented by aucubin treatment, and IR-induced nuclear translocation of NF-κB was reversed by aucubin treatment. Briefly, aucubin exhibited neuroprotective effects against brain IR injury, which might be related to the attenuation of neuroinflammation through inhibiting the TLR-4/NF-κB signaling pathway. These results suggest that aucubin pretreatment may be a potential approach for the protection of brain IR injury.

## 1. Introduction

Transient global cerebral ischemia or IR is a medical emergency that occurs due to transient diminution in blood flow to the brain, typically during adverse events, such as cardiac arrest, shock, and asphyxiation [1,2]. In both humans and experimental animals, this event leads to irreversible and persistent damage in specific brain regions, including the hippocampus, ultimately resulting in neurological deficits [3,4]. The cornu ammonis 1 (CA1) field of the hippocampus is one of cerebral structures vulnerable to cerebral IR injury [5,6], resulting in a marked loss of principal (pyramidal) neurons located in this field several days after cerebral IR [7,8]. Pathological processes contributing to cerebral IR injury include glutamate-dependent excitotoxicity, excessive reactive oxygen species-induced oxidative stress, and reactive glial cells-mediated neuroinflammation [9,10,11]. Therefore, mitigating the processes may be one of the most crucial approaches for the prevention and treatment of cerebral IR injury.

Iridoid glycosides are a group of bioactive compounds commonly found in traditional medicinal plants, and they display a wide range of biological and pharmacological effects, including antioxidative, anti-inflammatory, hepatoprotective, and nephroprotective activities [12,13,14]. However, their potential neuroprotective activities have been rarely studied. Aucubin (AU) is an iridoid glycoside isolated from *Aucuba japonica* and *Veronica persica* [15,16]. Previous experimental studies have shown that AU effectively prevents the loss of pyramidal neurons (principal neuronal cells) in the hippocampal CA1 field in rats with diabetic encephalopathy [17,18]. This neuroprotective effect is closely associated with the antioxidant activity of AU [18]. Recently, we also reported that AU protected hippocampal CA1 pyramidal neurons from IR injury in gerbils through its antioxidant activity [19]. However, whether AU can display neuroprotective effects by protecting and/or attenuating neuroinflammation remains to be fully explained. Therefore, the purpose of this experiment was to investigate whether AU attenuated cerebral IR-induced neuroinflammation and whether its anti-inflammatory activity contributed to neuroprotection against the cerebral IR injury using gerbils subjected to cerebral IR. Additionally, we explored whether the anti-inflammatory activity of AU was related to the downregulation of the toll-like receptor 4 (TLR4)/nuclear factor-κB (NF-κB) signaling pathway, which is a major pathway involved in the neuroinflammatory response induced by cerebral IR [20,21]. 

## 2. Results

### 2.1. Protection of Cerebral IR-Induced Neuronal Death by AU

#### 2.1.1. NeuN-Immunoreactive (NeuN^+^) Neurons

**Figure 1:**
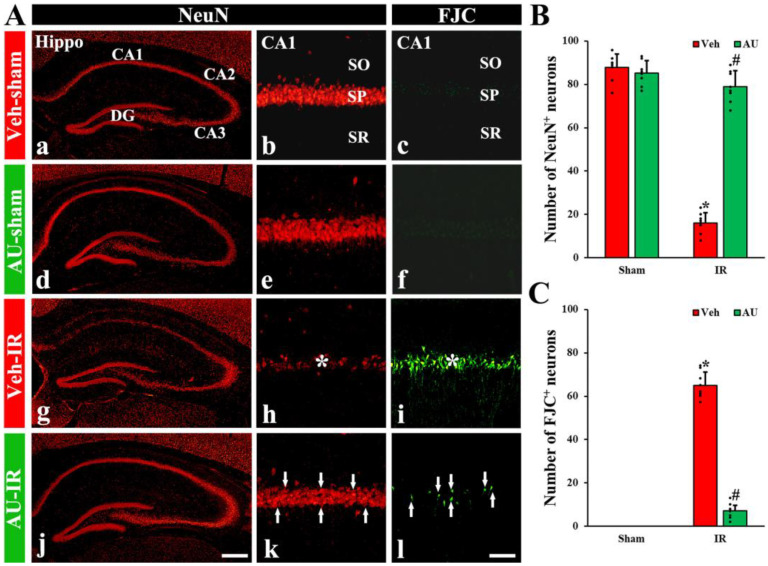
(**A**) Representative photomicrographs of NeuN immunofluorescence (**left** and **middle** panels) and FJC fluorescence (**right** panels) in the hippocampal CA1 field of the Veh-sham (**a**–**c**), AU-sham (**d**–**f**), Veh-IR (**g**–**i**), and AU-IR (**j**–**l**) groups on day 4 after cerebral IR. In the Veh-IR group, a few NeuN^+^ (red) and numerous FJC^+^ (green) neurons are shown in the stratum pyramidale (SP, asterisks in (**h**,**i**)). However, in the AU-IR group, an increased number of NeuN^+^ neurons and a decreased number of FJC^+^ neurons are found in the SP (arrows in (**k**,**l**)). CA, cornu ammonis; DG, dentate gyrus; SO, stratum oriens; SR, stratum radiatum. Scale bar = 400 µm (**a**,**d**,**g**,**j**), 60 µm (**b**,**c**,**e**,**f**,**h**,**i**,**k**,**l**). (**B**,**C**) Quantification of NeuN^+^ (**B**) and FJC^+^ neurons (**C**) in the CA1 field. The error bars represent mean ± SD (*n* = 8/group; * *p* < 0.05 vs. corresponding sham group, ^#^ *p* < 0.05 vs. Veh-IR group).

Immunofluorescence staining with anti-NeuN (a mature neuronal marker) was performed to examine neuronal survival in the hippocampal CA1 field on day 4 after cerebral IR. In the vehicle (Veh)-sham group (treated with saline and subjected to sham IR) and AU-sham groups, NeuN immunofluorescence was prominently shown in pyramidal neurons, which constitute the stratum pyramidale in all subfields (CA1–3) of the hippocampus (Figure 1A(a,b,d,e)). No significant difference was observed in the number of NeuN^+^ pyramidal neurons in the CA1 field between the two groups (Figure 1B). In the Veh-IR group (treated with saline and subjected to cerebral IR), a significant diminution in the number of NeuN^+^ pyramidal neurons (16 ± 4.3 neurons/300 × 300 μm^2^) was found in the CA1 field (Figure 1A(g,h),B). However, in the AU-IR group, a substantial number of NeuN^+^ CA1 pyramidal neurons (79 ± 7.4 neurons/300 × 300 μm^2^) were detected, which was significantly high in number when compared with the Veh-IR group (Figure 1A(j,k),B).

#### 2.1.2. FJC-Positive (FJC^+^) Neurons

Histofluorescence staining with FJC (a marker of degenerating or dead neurons) was performed to detect degenerating or dead neurons in the hippocampal CA1 field on day 4 after cerebral IR. In the Veh-sham and AU-sham groups, No FJC^+^ neurons were detected in the CA1 field (Figure 1A(c,f)). In the Veh-IR group, numerous FJC^+^ pyramidal neurons (65 ± 6.2 neurons/300 × 300 μm^2^) were observed in the CA1 field (Figure 1A(i),C). However, in the AU-IR group, only a few FJC^+^ CA1 pyramidal neurons (7 ± 2.5 neurons/300 × 300 μm^2^) were found (Figure 1A(l),C).

### 2.2. Attenuation of Cerebral IR-Induced Gliosis by AU

#### 2.2.1. Microgliosis

**Figure 2:**
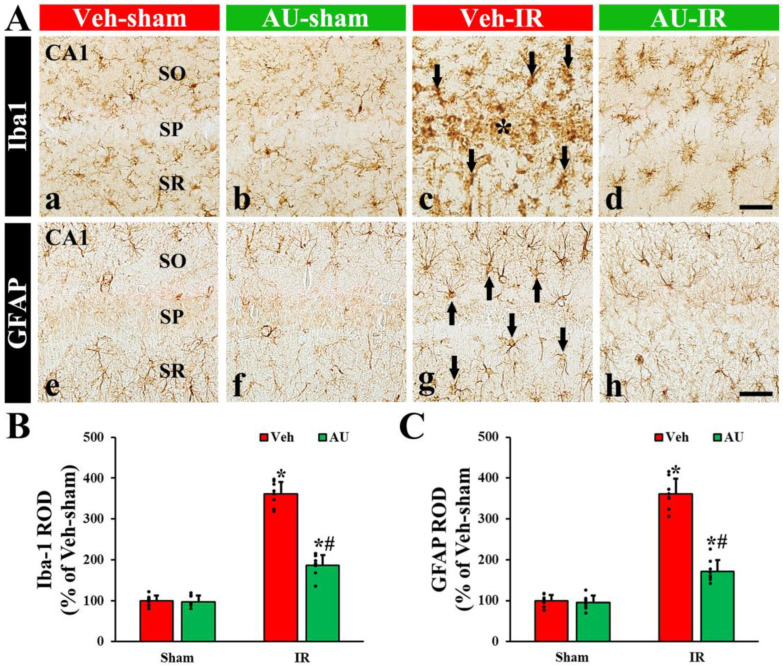
(**A**) Representative photomicrographs of Iba1 (**upper** panels) and GFAP immunohistochemistry (**lower** panels) in the CA1 field of the Veh-sham (**a**,**e**), AU-sham (**b**,**f**), Veh-IR (**c**,**g**), and AU-IR (**d**,**h**) groups at 4 days after cerebral IR. No significant differences in the morphology of Iba1^+^ and GFAP^+^ glial cells are observed between the Veh-sham and AU-sham groups. In the Veh-IR group, reactive Iba1^+^ and GFAP^+^ glial cells (arrows in (**c**,**g**)) are distinctly observed: especially, many reactive Iba1^+^ glial cells are concentrated in the stratum pyramidale (SP, asterisk in (**c**)). However, in the AU-IR group, the reaction of Iba1^+^ and GFAP^+^ glial cells is apparently weakened as compared to that of the Veh-IR group. SO, stratum oriens; SR, stratum radiatum. Scale bar = 60 µm. (**B**,**C**) Quantitative analysis of Iba1^+^ (**B**) and GFAP^+^ (**C**) structures in the CA1 field. The error bars represent mean ± SD (*n* = 8/group; * *p* < 0.05 vs. corresponding sham group, ^#^ *p* < 0.05 vs. Veh-IR group).

To evaluate microglial reaction (or response) called microgliosis in the CA1 field on day 4 after cerebral IR, immunohistochemistry with anti-Iba1 (a maker of microglia or microglial cells) was conducted. In the Veh-sham and AU-sham groups, Iba1^+^ microglia exhibited small and round cell bodies with long and thin processes, indicating a resting state, and they were scattered throughout the CA1 field (Figure 2A(a,b)). There was no significant difference in the relative optical density (ROD), as % Iba1 immunoreactivity, of Iba1^+^ microglia between the two groups (Figure 2B). In the Veh-IR group, Iba1^+^ microglia displayed a reactive state with hypertrophic cell bodies and stouter processes, which were concentrated in the stratum pyramidale (Figure 2A(c)). In this group, the ROD of Iba1^+^ was significantly higher (452% of the Veh-sham) than that of the Veh-sham group (Figure 2B). In contrast, the reaction of Iba1^+^ immunoreactive microglia in the AU-IR group was significantly attenuated (Figure 2A(d)): the ROD of the Iba1^+^ immunoreactive microglia was 41% of the Veh-IR group (Figure 2B).

#### 2.2.2. Astrogliosis

To examine the astrocyte reaction called astrogliosis in the CA1 field on day 4 after cerebral IR, immunohistochemistry was conducted with anti-glial fibrillary acidic protein glia (GFAP, a marker of astrocyte). In the Veh-sham and AU-sham groups, GFAP^+^ astrocytes possessed small cell bodies and thread-like processes, as resting astrocytes, and they were ubiquitously present throughout the CA1 field (Figure 2A(e,f)). No significant difference was observed in the ROD of GFAP^+^ astrocytes between the two groups (Figure 2C). In the Veh-IR group, GFAP^+^ astrocytes were hypertrophied, with enlarged cell bodies and thickened processes, as reactive astrocytes (Figure 2A(g)). In this group, the ROD was significantly increased (361% of the Veh-sham) when compared with that of the Veh-sham group (Figure 2C). However, in the AU-IR group, the hypertrophy of GFAP^+^ immunoreactive astrocytes was markedly decreased (Figure 2A(h)), showing that the ROD was significantly lower (48% of the Veh-IR) than that of the Veh-IR group (Figure 2C). 

### 2.3. Attenuation of Cerebral IR-Induced Increases of Proinflammatory Cytokines by AU

**Figure 3:**
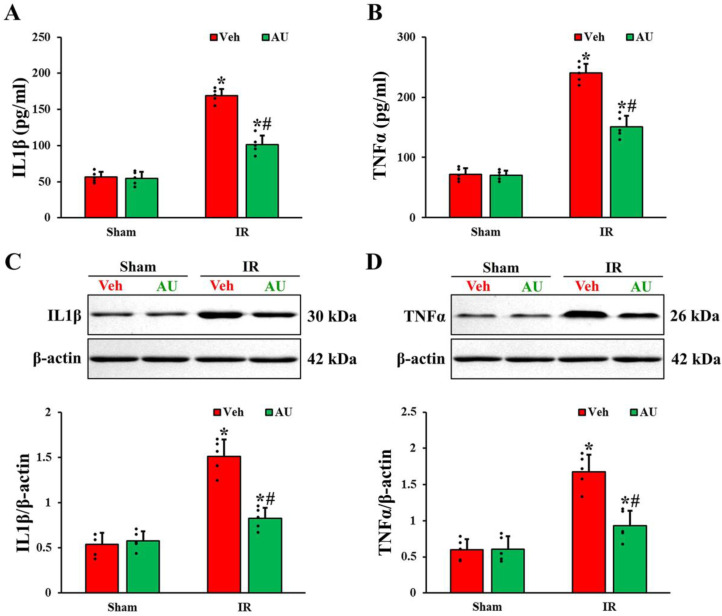
(**A**,**B**) Levels of IL1β (**A**) and TNFα (**B**) in the serum of the Veh-sham, AU-sham, Veh-IR, and AU-IR groups at 1 day after cerebral IR. (**C**,**D**) Western blot bands (*n* = 1 per lane) and quantitative analyses of IL1β (**C**) and TNFα (**D**) in the CA1 field extracted from the Veh-sham, AU-sham, Veh-IR, and AU-IR groups at 1 day after IR. Note that Western blot bands for *n* = 4 are included in the Supplementary Materials Figures S1–S4. IL1β and TNFα levels in both serum and CA1 field of the Veh-IR group are enhanced, but those in the AU-IR group are low as compared to those of the Veh-IR group. The error bars represent mean ± SD (*n* = 5/group; * *p* < 0.05 vs. corresponding sham group, ^#^ *p* < 0.05 vs. Veh-IR group).

To evaluate the levels of proinflammatory cytokines, IL1β and TNFα, in the serum and hippocampal CA1 field on day 1 after cerebral IR, ELISA and Western blot analyses were performed. As shown in Figure 3A,B, the serum levels of IL1β and TNFα in the AU-sham group were not different from those evaluated in the Veh-sham. In the Veh-IR group, the levels of serum IL1β and TNFα were increased (3 times and 3.3 times, respectively, higher than those in the Veh-sham group). However, the levels of serum IL1β and TNFα in the AU-IR group were decreased by 40% and 37%, respectively, when compared with those in the Veh-IR group. 

The protein levels of IL1β and TNFα in the CA1 field were consistent with those in the serum (Figure 3C,D). In the Veh-sham and AU-sham groups, no significant differences were shown in the protein levels of IL1β and TNFα. In the Veh-IR group, IL1β and TNFα protein levels were increased (2.8 times and 2.9 times, respectively, higher than those evaluated in the Veh-sham group). However, in the AU-IR group, IL1β and TNFα protein levels were lower by 45% and 46%, respectively, as compared with the Veh-IR group. 

### 2.4. Inactivation of Transient Global Cerebral IR-Mediated TLR4/NF-κB Signaling Pathway by AU

**Figure 4:**
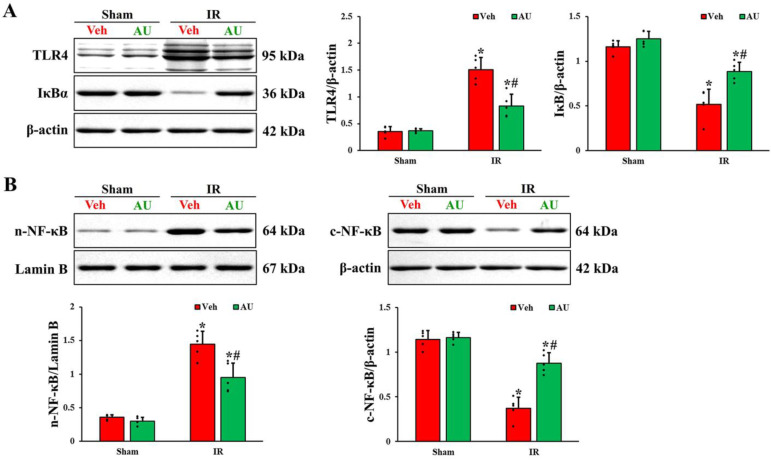
(**A**) Western blot bands (*n* = 1 per lane) and quantitative analyses of TRL4 and IκBα in the CA1 field extracted from the Veh-sham, AU-sham, Veh-IR, and AU-IR groups at 1 day after cerebral IR. IR upregulates TLR4 and downregulates IκBα, but AU treatment prevents it. (**B**) Western blot bands (*n* = 1 per lane) and quantitative analyses of NF-κB p65 in the nuclear and cytoplasmic fractions of the CA1 field obtained from the Veh-sham, AU-sham, Veh-IR, and AU-IR groups at 1 day after cerebral IR. IR apparently induces the nuclear-cytoplasmic translocation of NF-κB p65, but AU mitigates it. Note that Western blot bands for *n* = 4 are included in the Supplementary Materials Figures S5–S12. The error bars represent mean ± SD (*n* = 5/group; * *p* < 0.05 vs. corresponding sham group, ^#^ *p* < 0.05 vs. Veh-IR group).

To investigate the possible mechanism of AU-mediated neuroprotection against cerebral IR injury, the levels of TLR4/NF-κB signaling pathway-related proteins were examined in the CA1 field on day 1 after cerebral IR using Western blotting. As shown in Figure 4A, no significant differences in the levels of TLR4 and IκBα between the Veh-sham and AU-sham groups were found. In the Veh-IR group, the TLR4 level was enhanced (4.2 times higher than that evaluated in the Veh-sham group), and the IκBα level was reduced (55% of the Veh-sham group) as compared to that in the Veh-sham group. However, in the AU-IR group, the IR-induced upregulation of TLR4 and downregulation of IκBα were prevented (50% and 169%, respectively, of the Veh-IR group). In addition, as shown in Figure 4B, the translocation of NF-κB p65 to the nuclei was observed (Figure 4B). There were no significant differences in the levels of NF-κB p65 in both nuclear and cytoplasmic fractions between the Veh-sham and AU-sham groups. The NF-κB p65 level in the nuclear fraction of the Veh-IR group was increased (4 times higher than that of the Veh-sham group), but the NF-κB p65 level in the cytoplasmic fraction concurrently decreased (67% of the Veh-sham group), indicating that NF-κB p65 translocated from the cytoplasm to the nucleus. However, the nuclear-cytoplasmic translocation of NF-κB p65 was mitigated (66% and 238%, in the nuclear fraction and cytoplasmic fraction respectively, of the Veh-IR group) in the AU-IR group.

## 3. Discussion

In a gerbil model of 5 min transient forebrain ischemia, CA1 pyramidal neuronal death accompanied by reactive gliosis (microgliosis and astrogliosis) occurred 4 to 5 days after induction of IR. For this reason, it is important to investigate changes in biomarkers including inflammatory cytokines by lapse of time after IR in order to study the mechanisms of the neuronal death and neuroprotection. Thus, we investigated CA1 pyramidal neuronal death and reactive gliosis at four days after IR and examined changes in pro-inflammatory cytokines and their related biomarkers involved in the TLR-4/NF-κB signaling pathway at 1 day after IR.

In recent decades, bioactive compounds derived from medicinal plants have gained significant attention for their diverse biological and pharmacological effects, as well as their safety aspects, in the prevention and treatment of neurological disorders [22]. Many experimental studies have demonstrated that these compounds exert beneficial effects against cerebral IR injury [23,24]. In this study, we examined the potential neuroprotective effects of AU in a gerbil model of cerebral IR using NeuN immunofluorescence and FJC histofluorescence staining and found that pretreatment with 10 mg/kg of AU, an iridoid glycoside, effectively protected pyramidal neurons, principal neuronal cells, in the hippocampal CA1 field from cerebral IR injury. This result is consistent with our previous study, which had been conducted in gerbils that received cerebral IR injury [19]. Some researchers have demonstrated that iridoid glycoside has neuroprotective effects against brain ischemic injury. For example, Whang et al. (2019) reported that cornel iridoid glycoside, a major active component extracted from *Cornus officinalis*, displayed protection on the white matter injury induced by brain IR in rats [25]. In addition, Lan et al. (2022) showed that cornin (an iridoid glycoside) derived from the fruit of *Verbena officinalis* L. significantly reduced cerebral infarction volume indued by focal IR in rats. Taken together, iridoid glycoside can be a powerful candidate for the protection against brain IR injury [26].

Accumulating evidence has revealed that glial cells play a pivotal role in cerebral IR injury [27,28,29]. Cerebral IR induces the reaction of microglia and astrocytes, as evidenced by the presence of enlarged cell bodies and hypertrophic processes, and they secrete diverse proinflammatory mediators, which contribute to severe neuroinflammatory responses and the aggravation of cerebral IR injury [30,31]. Thus, inhibiting the reaction of microglia and astrocytes has been considered as a part of neuroprotection against cerebral IR injury [9]. In this study, Iba1^+^ microglia and GFAP^+^ astrocytes in the Veh-IR group displayed a strong reaction with hypertrophic cell bodies and stouter processes in the CA1 filed, and, in particular, numerous Iba1^+^ microglia were concentrated in the stratum pyramidale. However, AU pretreatment significantly attenuated IR-induced strong reaction of the microglia and astrocytes. Wang et al. (2012) reported that geniposide, an iridoid glycoside isolated from Gardenia, displayed a neuroprotective effect against IR injury in a rat model of transient focal brain ischemia and inhibited oxygen–glucose deprivation (OGD)-induced reaction (activation) of microglial cells [32]. Taken together, iridoid glycoside has an efficacy inhibiting IR-induced reaction of glial cells.

IL1β and TNFα are well-recognized proinflammatory cytokines that modulate cerebral IR injury [33]. It has been reported that the levels of IL1β and TNFα, with the reaction of microglia and astrocytes, significantly increase in both brain tissue and serum after cerebral IR, and this increase is strongly related to the severity of ischemic brain damage [34,35]. In addition, studies have demonstrated that the pharmacological reduction of IL1β and TNFα levels is closely associated with neuroprotection against cerebral IR injury [36,37]. Wang et al. (2012) showed that OGD increased the release of TNFα and IL1β and the effect was suppressed by an iridoid glycoside (geniposide) [32]. In this study, IL1β and TNFα levels in the CA1 field and serum of the Veh-IR group were significantly increased after IR when compared with those of the Veh-sham group, but the IR-induced increase of the IL1β and TNFα levels was significantly decreased by AU pretreatment. Therefore, our results suggest that AU pretreatment suppresses the release of proinflammatory cytokines caused by cerebral IR. As this finding is the first, AU has anti-inflammatory activity in cerebral IR injury, implying that the activity of AU may contribute to neuroprotection against cerebral IR injury.

TLR4, as a key inflammatory mediator related to cerebral IR injury, is primarily expressed in microglia and astrocytes in ischemic brains [38,39]. Its activation is initiated through the binding of endogenous ligands that are released from stressed or damaged cells following cerebral IR and results in the nuclear translocation and activation of NF-κB, which promotes the release of proinflammatory cytokines and ultimately leads to ischemic neuronal death [40]. It has been demonstrated that cerebral IR injury is significantly decreased in TLR4-deficient mice [41,42]. Additionally, inhibiting the TLR4/NF-κB signaling pathway has shown the significant attenuation of neuroinflammatory response and cerebral IR injury [43,44]. In our current study, the treatment with AU significantly diminished the cerebral IR-induced upregulation of TLR4 and downregulation of IκBα (an inhibitor protein preventing nuclear transport and activation of NF-κB) and significantly reversed the nuclear translocation of NF-κB in the ischemic CA1 field. This result is supported by Wang et al. (2012), who reported that geniposide (an iridoid glycoside) attenuated the increases in OGD-induced TLR4 mRNA [32]. In addition, Zhang et al. (2020) recently reported that AU significantly reduced the inflammatory response induced by liver IR through the inhibition of the TLR4/NF-κB signaling pathway [45]. Taken together, it seems that AU treatment ameliorates the neuroinflammatory response caused by cerebral IR, possibly through inhibiting the TLR4/NF-κB signaling pathway. 

This study has several limitations. First, the gerbil model of 5 min transient forebrain ischemia probably does not perfectly incarnate IR injury shown in human brains because gerbils have a different cerebrovascular system from that of humans: the gerbils lack the posterior communicating arteries. Second, in order to identify exact molecular works of neuroprotective effects, it is important to verify potential downstream effects attributed by the TLR-4/NF-κB signaling pathway using siRNA. Therefore, for comprehensive and insightful studies, we suggest that, in follow-up studies, neuroprotective effects of AU should be investigated using a clinically significant animal model of ischemic stroke. Moreover, in vitro experiments using siRNA need to verify potential downstream mechanisms underlying the anti-inflammatory action of AU.

In summary, the present study revealed that pretreatment with AU protected hippocampal CA1 pyramidal neurons from cerebral IR injury, showing that AU treatment prevented the IR-induced increase in proinflammatory cytokines (IL1β and TNFα) and IR-induced upregulation of TLR4 and downregulation of IκBα, and reversed the IR-induced nuclear translocation of NF-κB. These indicate that AU pretreatment might exert anti-inflammatory activity, thereby providing neuroprotective effects against cerebral IR injury through inhibiting the TLR4/NF-κB signaling pathway. Therefore, our findings offer valuable insights suggesting that AU can be a promising candidate for the prevention of cerebral IR injury.

## 4. Materials and Methods

### 4.1. Ethical Statement and Experimental Animals

Adult male gerbils weighing 65 ± 5 g (at 7 months of age) bred in the animal house of the Experimental Animal Center (an affiliate of Kangwon National University) were used. The animal house was controlled at a temperature of 23 ± 2 °C, 56 ± 5% humidity, and 12:12 light/dark cycle. The procedures of all experiments were approved (approval number, KW-200113-1) on 18 February 2020 by the Institutional Animal Care and Use Committee. All efforts were carried out to minimize pain and/or distress in the animals throughout the entire experiment. In particular, the number of animals used for this experiment was minimized.

### 4.2. Experimental Groups, AU Treatment, and Induction of Cerebral IR

A total of 52 gerbils were randomly allocated to four groups (*n* = 13/group): (1) vehicle (Veh)-sham group, treated with saline (vehicle) and subjected to sham surgery; (2) AU-sham group, treated with 10 mg/kg of AU and subjected to sham surgery; (3) Veh-IR group, treated with saline and subjected to cerebral IR; and (4) AU-IR group, treated with 10 mg/kg of AU and subjected to cerebral IR. The dose of aucubin was chosen in accordance with our previous study [19].

**Figure 5:**
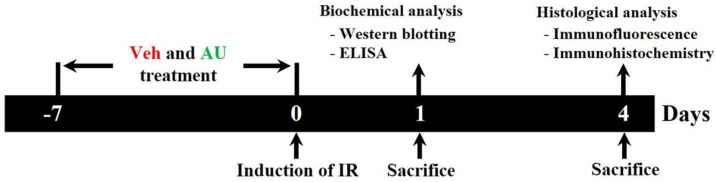
Schematic diagram illustrating the experimental procedure of AU treatment and the neuroprotective effect and mechanism of AU against transient global cerebral IR injury. Gerbils are subjected to transient global cerebral IR. AU or Veh is intraperitoneally injected once daily for 7 days before the induction of transient global cerebral IR. At 1 and 4 days after transient global cerebral IR, the gerbils are sacrificed, and brain samples are harvested for experimental analyses. AU, aucubin; ELISA, enzyme-linked immunosorbent assay; IR, ischemia and reperfusion; Veh, vehicle.

To inject 10 mg/kg of AU, AU (≥98% purity) was obtained from Sigma-Aldrich (St. Louis, MO, USA) and dissolved in saline. The dose and duration of AU treatment were determined based on the results of our previous study [19]. As shown in Figure 5, the gerbils were intraperitoneally treated with either AU or Veh once a day for 7 consecutive days before the induction of cerebral IR operation.

As previously described [37], cerebral IR was induced as follows. Briefly, the gerbils were adequately anesthetized with isoflurane (2.5%; JW Pharmaceutical Corporation, Seoul, Republic of Korea) using an inhalation anesthesia machine obtained from Harvard Apparatus (Holliston, MA, USA). Body (rectal) temperature was controlled at normothermia (37.0 ± 0.5 °C) for 30 min before and during the IRI surgery using a heating pad, which was connected to rectal thermistors, a homeothermic monitoring system (Harvard Apparatus, Holliston, MA, USA). To induce cerebral IR, a middle-neck incision was made, and both common carotid arteries, which supply blood to the brain, were isolated and blocked by ligation of the arteries using aneurysm clips for 5 min. The block of blood supply was confirmed: no circulation was observed in retinal arteries (branches of carotid arteries) with the ophthalmoscope HEINE K180 (Heine Optotechnik, Herrsching, Germany). Immediately, the clips were removed, and cerebral blood flow was allowed to re-establish. Thereafter, to prevent hypothermia, the gerbils were kept for 3 h in a thermal incubator (temperature of 23 ± 2 °C and 56 ± 5% humidity) to adjust body temperature (normothermic level). The gerbils of the sham group underwent a similar procedure, except the common carotid arteries were occluded. During the experimental procedure, no significant change in physical health condition and body weight of the gerbils was found.

### 4.3. Tissue Preparation for Histological Analysis

For histological analysis, 8 gerbils in each group were sacrificed on day 4 after cerebral IR (Figure 5). In brief, as previously described by [46], the gerbils were deeply anesthetized with urethane (1.5 g/kg, intraperitoneally; Sigma-Aldrich, St. Louis, MO, USA) and received transcardial perfusion of 50 mM phosphate-buffered saline (PBS, pH 7.4) to wash the brains followed by 4% paraformaldehyde for the fixation of the brains. Subsequently, the brains were carefully extracted from the skulls and postfixed with 4% paraformaldehyde for 10 h. Thereafter the brains were soaked in 30% sucrose for cryoprotection. To make histological sections, the brain tissues including the hippocampi were serially sectioned into 30 μm thick coronal planes using Leica freezing microtome (Wetzlar, Germany).

### 4.4. NeuN Immunofluorescence and FJC Histofluorescence Staining

To evaluate neuronal death and/or survival in the CA1 field on day 4 after cerebral IR (Figure 5), we performed immunofluorescence with anti-NeuN (a mature neuronal marker) and histofluorescence staining with FJC (a marker of degenerating neurons). As previously described by [37], in short, for NeuN immunofluorescence staining, the sections were incubated in mouse anti-NeuN (diluted at 1:800; Merck Millipore, MA, USA) for 9 h at 4 °C, washed briefly, incubated in Cy3-conjugated donkey anti-mouse immunoglobulin G (IgG, diluted at 1:400; Vector Laboratories, Burlingame, CA, USA) for 2 h at room temperature. For FJC histofluorescence staining, the sections were immersed in 1% sodium hydroxide and 0.06% potassium permanganate for 15 min, respectively, and finally in 0.0004% FJC (Biosensis, Thebarton, SA, Australia) for 20 min.

NeuN^+^ and FJC^+^ neurons were counted using a method by [37]. Briefly, 5 sections/gerbil were selected at 120 μm intervals. The digital images of the CA1 field were acquired using BX53 fluorescence microscope (Olympus, Tokyo, Japan) with green (510–560 nm; for NeuN) and blue (450–490 nm; for FJC) excitation lights. The digital images of the NeuN^+^ and FJC^+^ neurons were obtained within a 300 × 300 μm^2^ including the stratum pyramidale at the center of the CA1 field. The cell counts were averaged using the image analysis system Optimas 6.5 (CyberMetrics, Scottsdale, AZ, USA).

### 4.5. Immunohistochemistry

To assess the gliosis (microgliosis and astrogliosis) in the CA1 field on day 4 after IR (Figure 5), immunohistochemistry was performed with anti-Iba1 for microglial cells and anti-GFAP for astrocytes. As described previously [47], in brief, the sections were incubated in rabbit anti-Iba1 (diluted at 1:800; Wako, Osaka, Japan) and rabbit anti-GFAP (diluted at 1:800; Chemicon, Temecula, CA, USA) for 8 h at 4 °C, washed briefly, and incubated in a secondary antibody of anti-rabbit IgG (diluted at 1:200; Vector Laboratories) for 2 h at room temperature. Continuously, the bound secondary antibody was amplified using an avidin–biotin complex kit (diluted at 1:200; Vector Laboratories) for 2 h at room temperature. Finally, the sections were visualized with 0.02% 3,3′-diaminobenzidine tetrahydrochloride (Sigma-Aldrich).

To evaluate the immunoreactivity of Iba1^+^ or GFAP^+^ structures, 5 sections/animal were selected. The digital images of the Iba1^+^ or GFAP^+^ structures were obtained using the aforementioned method. For the evaluation of each immunoreactivity, according to a method used by Kim et al. (2019) [47], the acquired images were calibrated to a 512 × 512 pixel array, and each immunoreactivity was measured using a 0–255 grayscale system. The ratio of the relative optical density (ROD) for the Iba1^+^ or GFAP^+^ structure was calibrated as a percentage (%) using Adobe Photoshop 8.0 (Adobe Systems, San Jose, CA, USA) and subsequently analyzed using ImageJ software version 1.59 (National Institutes of Health, Bethesda, MD, USA). The ROD ratio was compared to the Veh-sham group, which was designated as 100%.

### 4.6. Enzyme-Linked Immunosorbent Assay (ELISA)

To evaluate the serum levels of proinflammatory cytokines on day 1 after cerebral IR (Figure 5), 5/group were sacrificed. ELISA assays were performed for IL1β and TNFα. In brief, according to the method used by [48], the animals were deeply anesthetized with urethane and blood samples were collected via orbital puncture. The blood samples (one pooled sample per group) were centrifuged at 4000 rpm for 10 min and the sera were collected. The levels of IL1β and TNFα in the sera were measured using an ELISA kit according to the protocol provided by the manufacturer (Abcam, Cambridge, UK).

### 4.7. Western Blotting

To investigate the TLR4/NF-κB inflammatory signaling pathway in the CA1 field, 5/group were sacrificed on day 1 after cerebral IR (Figure 5). Western blotting was conducted using the method described by [10]. In short, the gerbils were sacrificed through decapitation after collecting blood samples, and their brains were immediately removed. The brains were then cut into 400 μm thick serial transverse sections using a vibratome. The tissues of the CA1 fields were carefully dissected from the brain slices using a surgical blade on ice plate. The samples of each gerbil were subsequently lysed in an ice-cold whole-cell lysate buffer. Nucleoprotein and cytoplasmic protein were extracted with the nuclear and cytoplasmic protein extraction kit obtained from Boster Biological Technology (Pleasanton, CA, USA). The protein concentration of each gerbil was determined using the colorimetric protein analysis kit of Bio-Rad (Hercules, CA, USA). Subsequently, 30 μg of protein was divided on sodium dodecyl sulfate-polyacrylamide gel and transferred to the nitrocellulose membrane obtained from Schleicher & Schuell GmbH (Dassel, Germany). Thereafter, it was blocked with 5% nonfat dry milk for 1 h at room temperature and incubated for 8 h at 4 °C with the primary antibodies: rabbit anti-IL1β (diluted at 1:1000; Abcam, Cambridge, UK), rabbit anti-TNFα (diluted at 1:1200; Abcam), rabbit anti-TLR4 (diluted at 1:1000; IMGENEX, San Diego, CA, USA), rabbit anti-NF-κB p65 (diluted at 1:1000; Abcam), rabbit anti-IκBα (diluted at 1:1000; Abcam), rabbit anti-Lamin B (diluted at 1:1000; Santa Cruz Biotechnology, Dallas, TX, USA), and rabbit anti-β-actin antibody (diluted at 1:5000; Sigma-Aldrich). The membranes were then exposed to peroxidase conjugated secondary antibody (diluted at 1:5000, Abcam) for 1 h at room temperature. The immunoreactive bands were visualized using the enhanced chemiluminescence reagent kit of Santa Cruz Biotechnology, and densitometric analysis was performed using ImageJ 1.59 software (National Institutes of Health, Bethesda, MA, USA).

### 4.8. Statistical Analysis

All of the data, in this research, were expressed as means ± standard deviation (SD). Statistical analysis was conducted using GraphPad Prism 5.0 of GraphPad Software (La Jolla, CA, USA). Significant differences between the groups were analyzed using one-way analysis of variance, followed by Bonferroni’s post hoc tests. The significant differences were determined in case the *p* value was less than 0.05.

## Data Availability

The data presented in this study are available on request from the corresponding author.

## Supplementary Materials

The supporting information can be downloaded at: https://www.mdpi.com/article/10.3390/ijms25063461/s1.
