# Supplementary material for: Neuroprotective Effects of Aucubin against Cerebral Ischemia and Ischemia Injury through the Inhibition of the TLR4/NF-κB Inflammatory Signaling Pathway in Gerbils
Source: Int J Mol Sci. 2024 Mar 19;25(6):3461. doi: 10.3390/ijms25063461 (PMC10970631; doi:10.3390/ijms25063461)
Supplement: Supplementary file 1 [file ijms-25-03461-s001.zip › ijms-2811144-supplementary.pdf]

Figure S1. IL1β

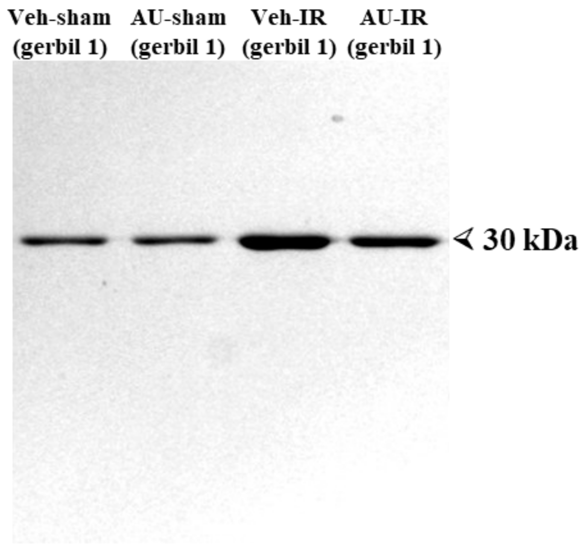

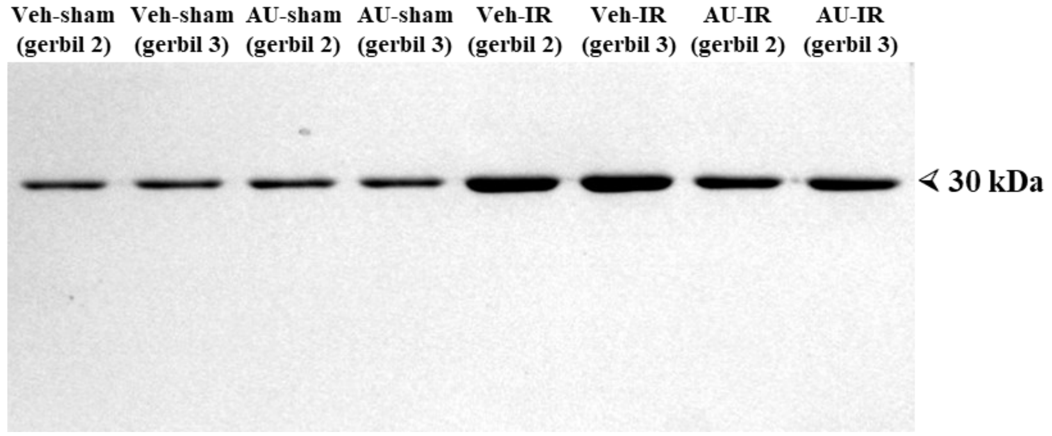

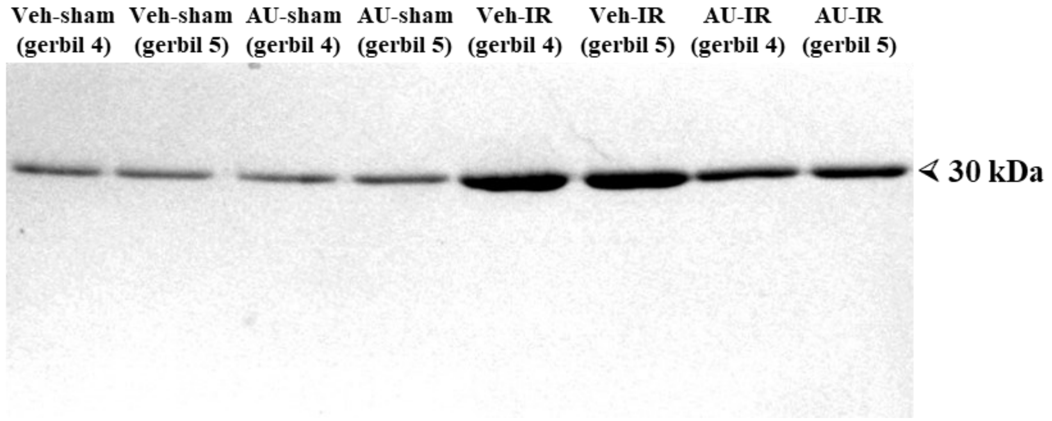

Figure S2. β-actin for IL1β

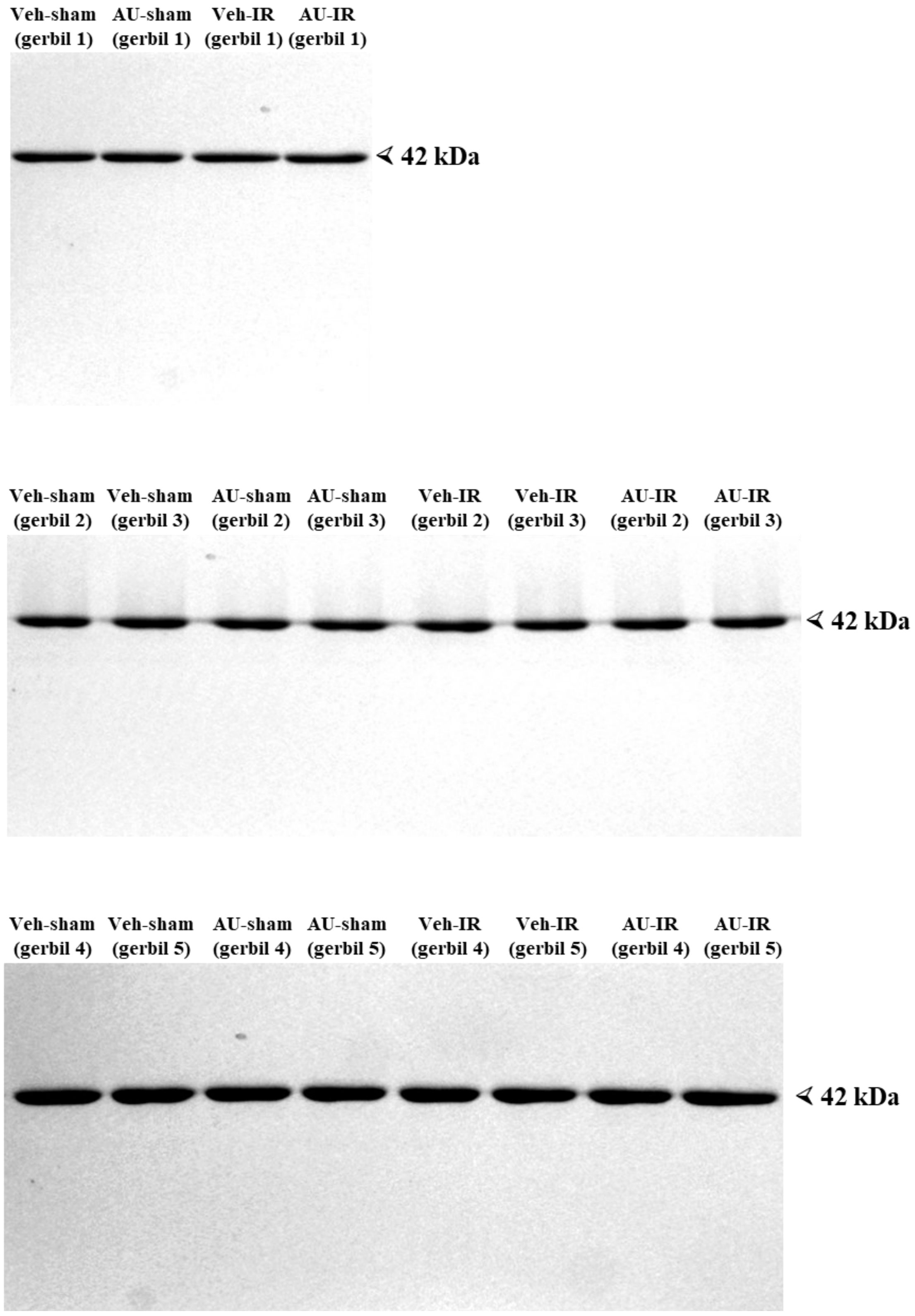

Figure S3. TNFα

Veh-sham (gerbil 1) | AU-sham (gerbil 1) | Veh-IR (gerbil 1) | AU-IR (gerbil 1)

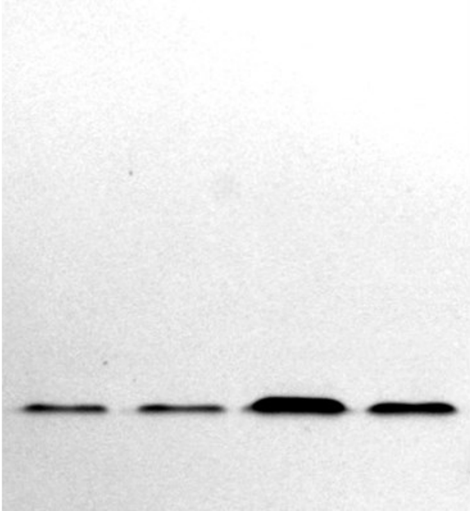

< 26 kDa

Veh-sham (gerbil 2) | Veh-sham (gerbil 3) | AU-sham (gerbil 2) | AU-sham (gerbil 3) | Veh-IR (gerbil 2) | Veh-IR (gerbil 3) | AU-IR (gerbil 2) | AU-IR (gerbil 3)

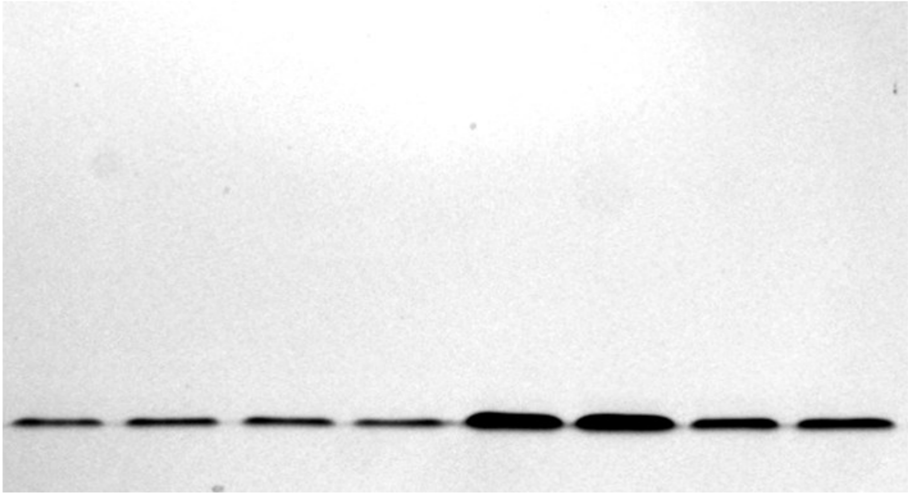

< 26 kDa

Veh-sham (gerbil 4) | Veh-sham (gerbil 5) | AU-sham (gerbil 4) | AU-sham (gerbil 5) | Veh-IR (gerbil 4) | Veh-IR (gerbil 5) | AU-IR (gerbil 4) | AU-IR (gerbil 5)

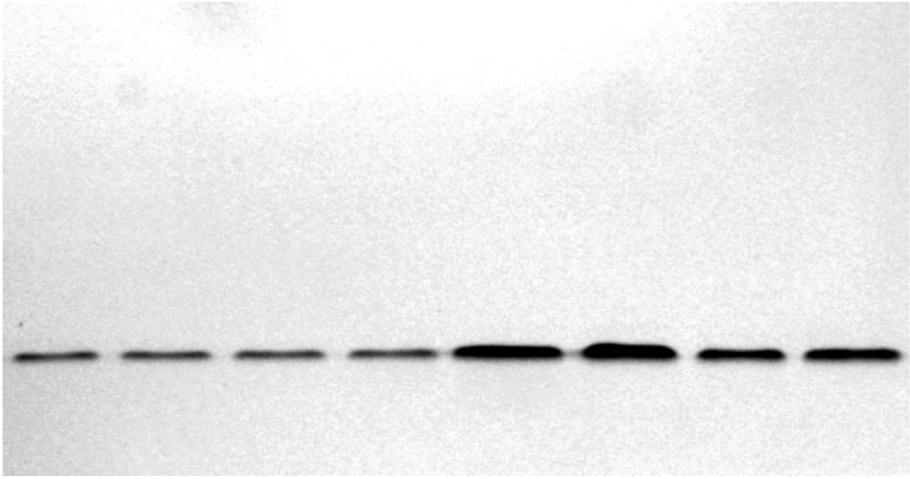

< 26 kDa

Figure S4. β-actin for TNFα

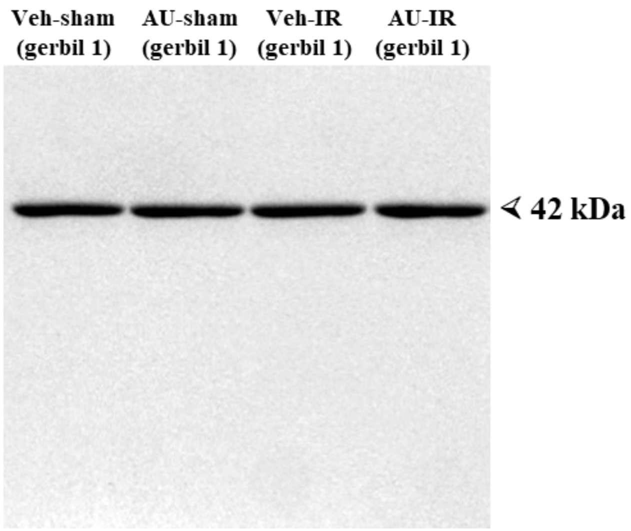

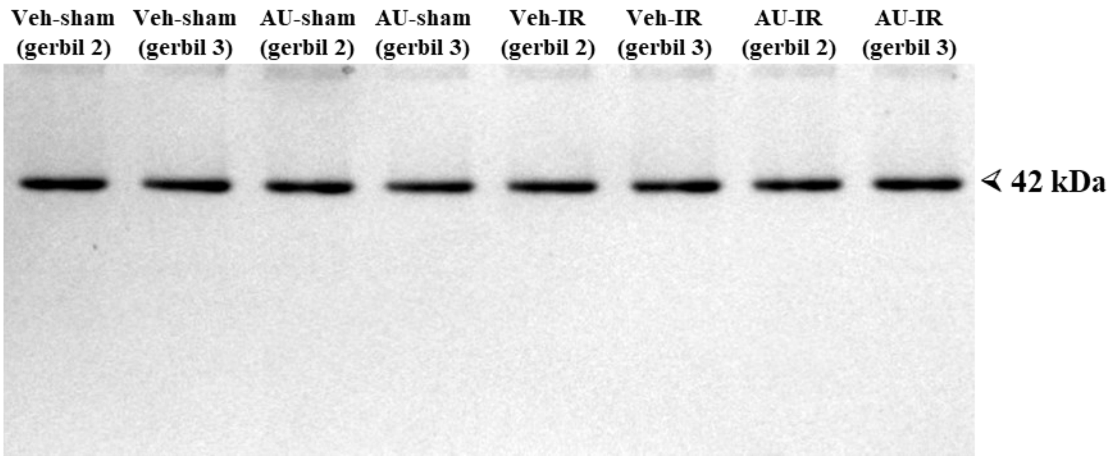

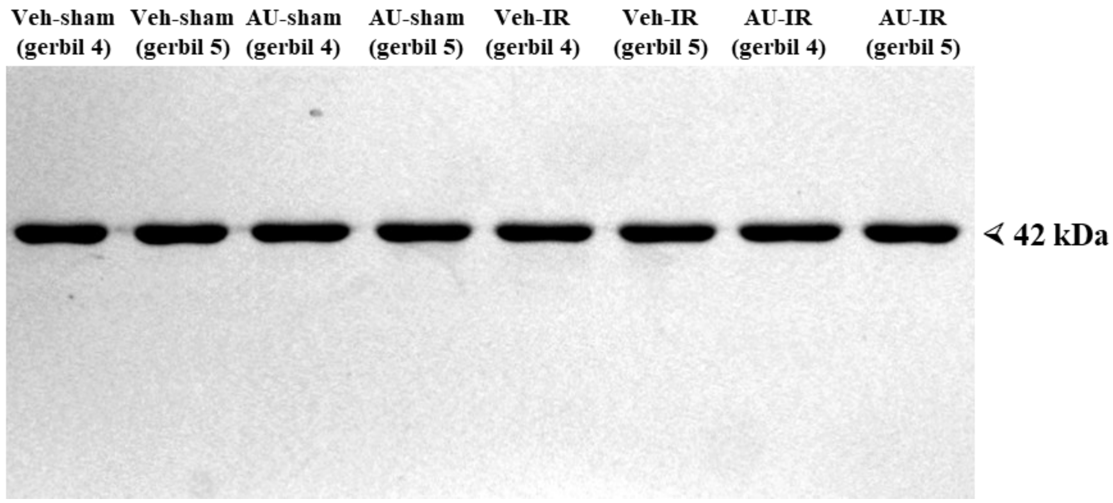

Supplementary Figures S1-4. Original images of Western blot data in Figure 3C,D

Figure S5. TLR4

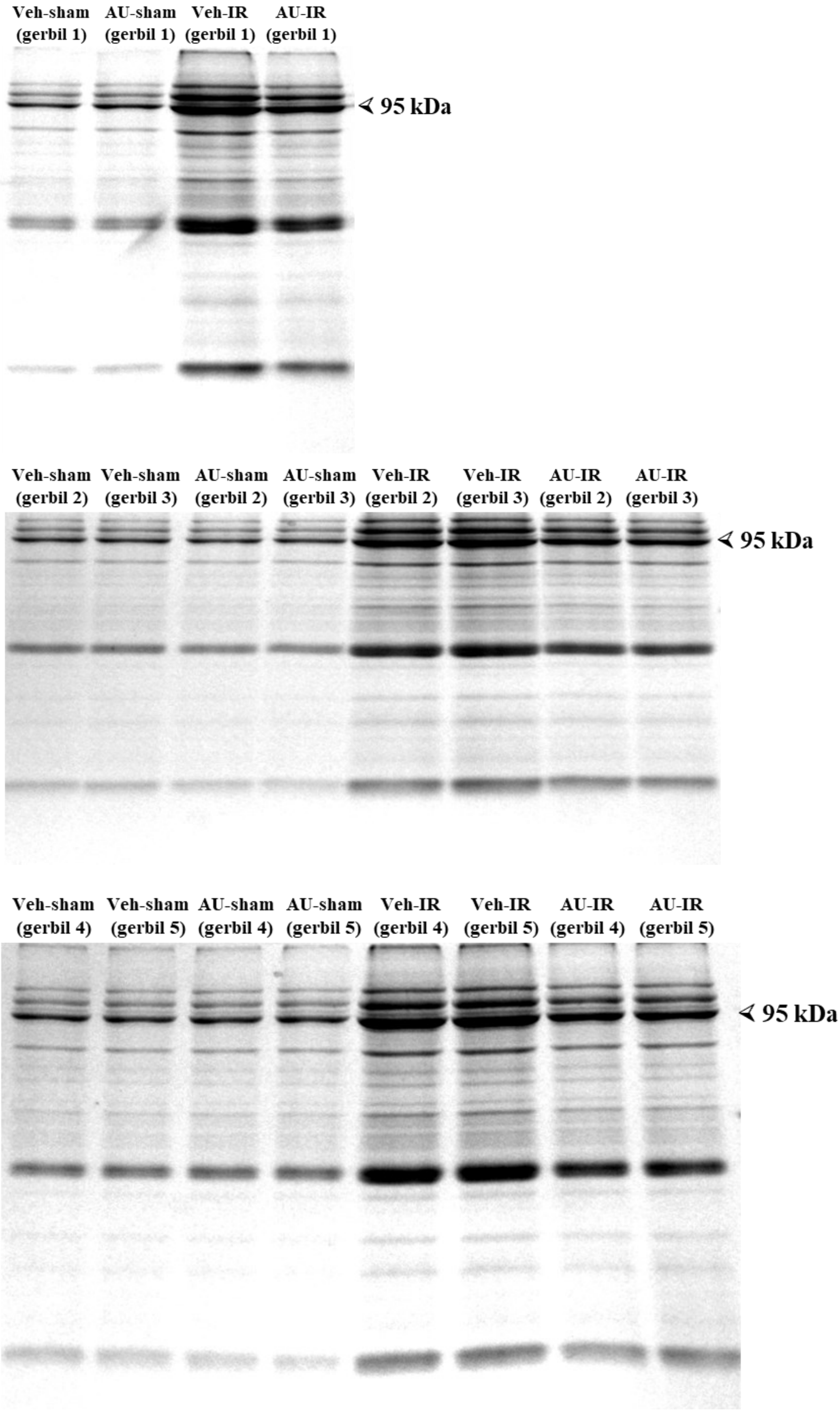

Figure S6. β-actin for TLR4

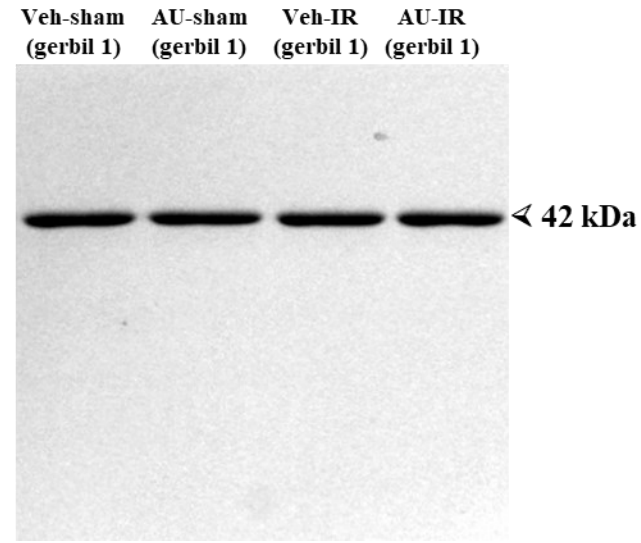

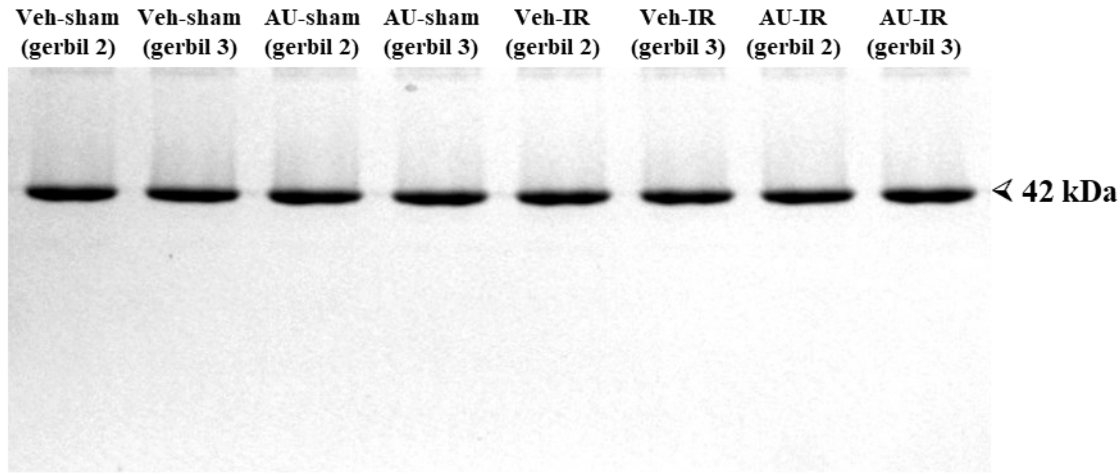

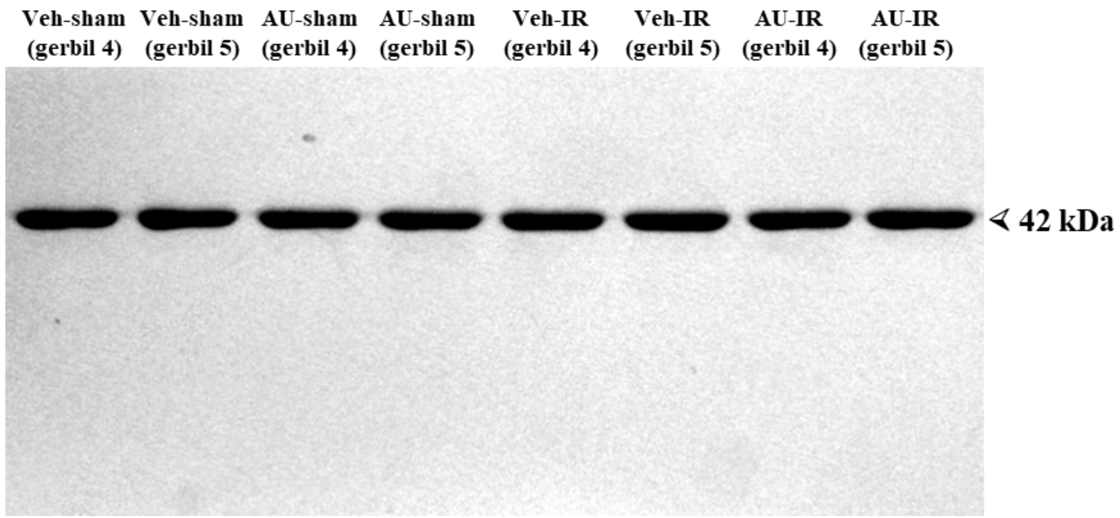

Figure S7. IκBα

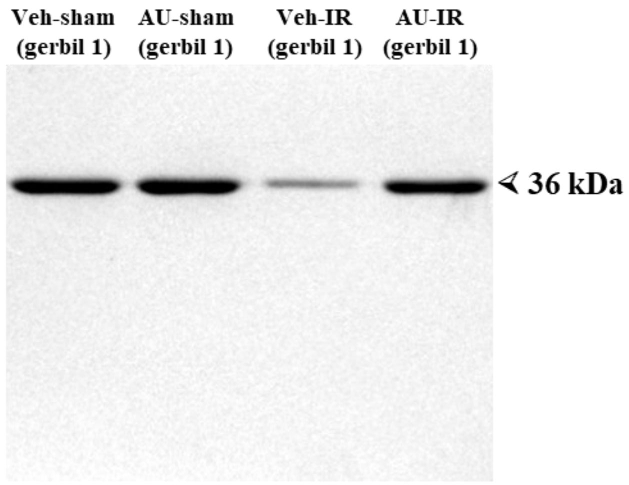

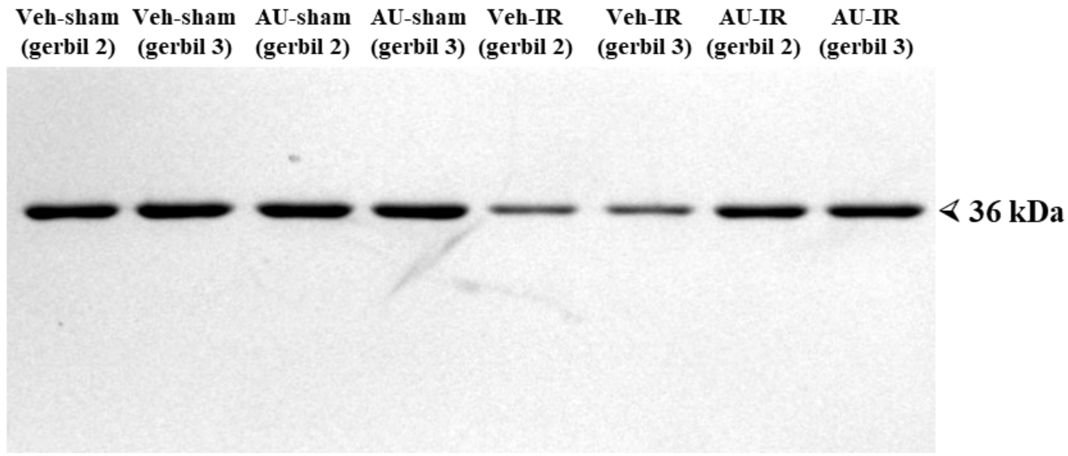

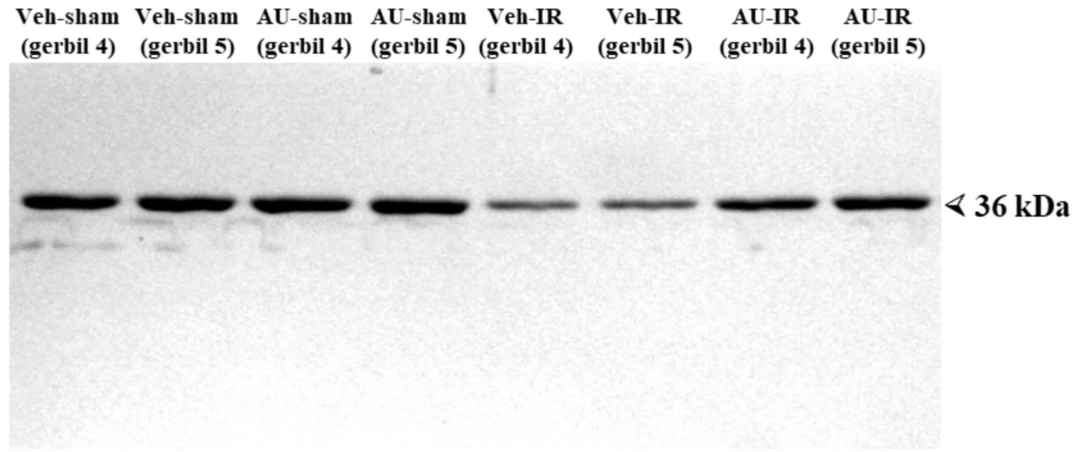

Figure S8. β-actin for IκBα

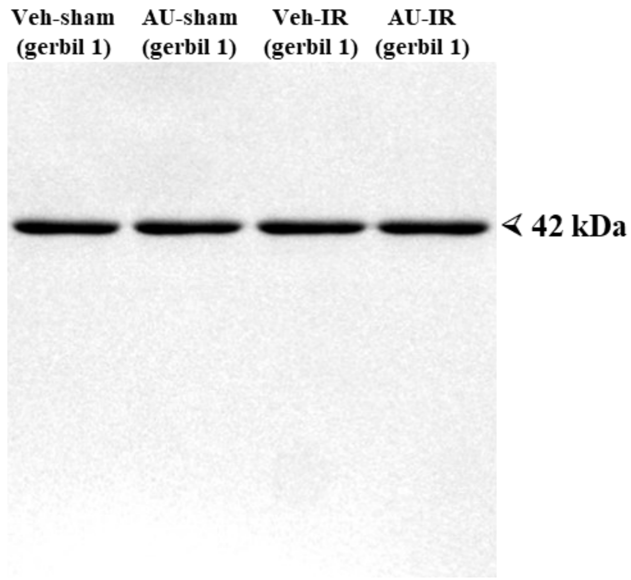

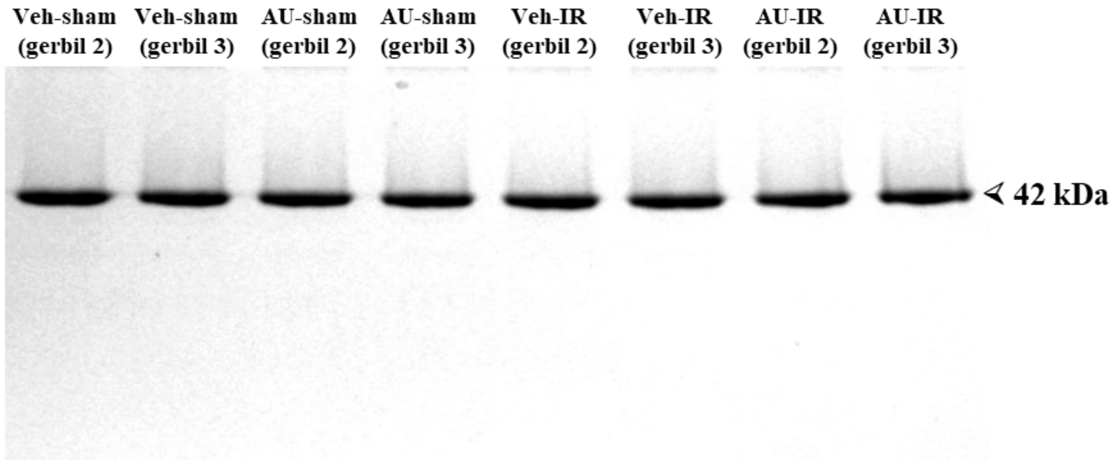

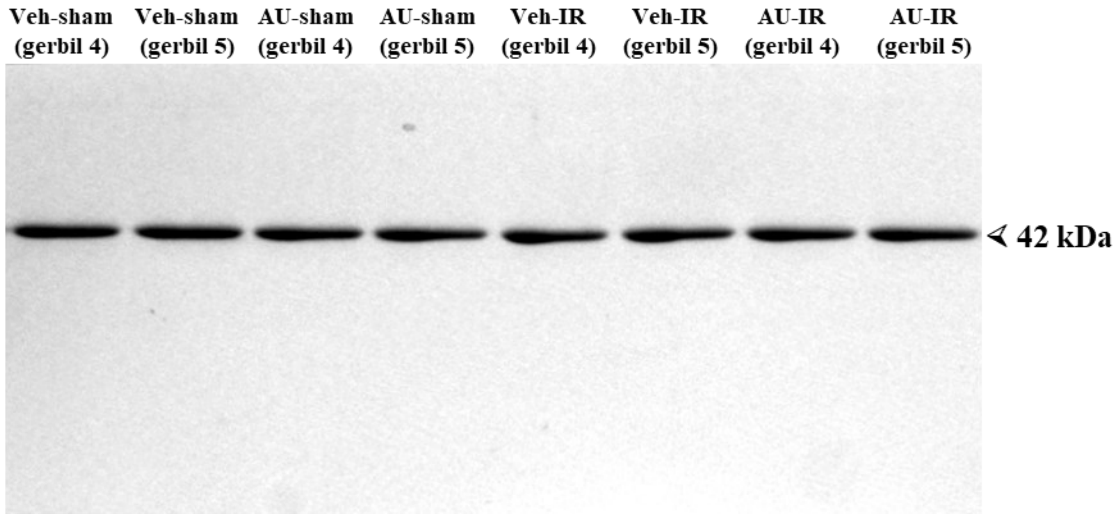

Figure S9. n-NF-κB

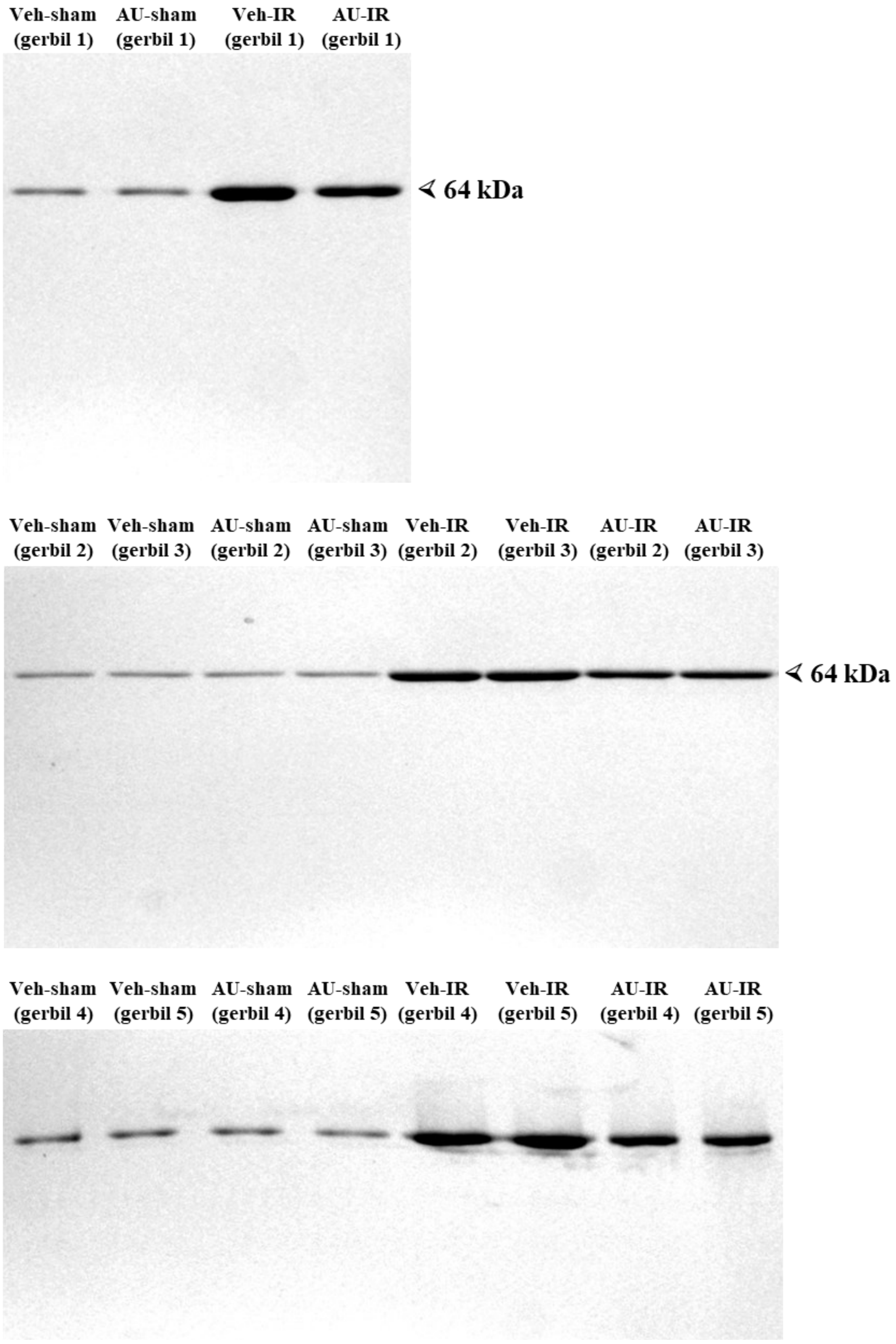

Figure S10. Lamin B for n-NF-κB

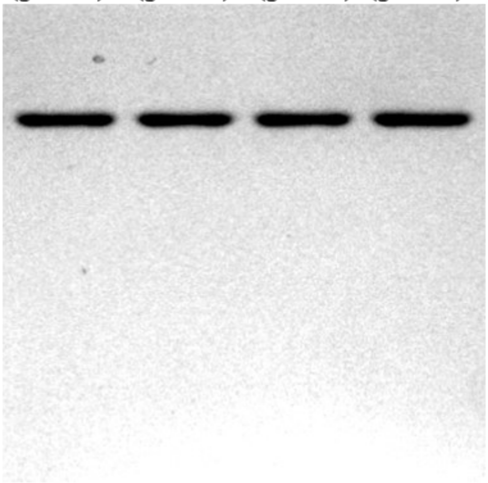

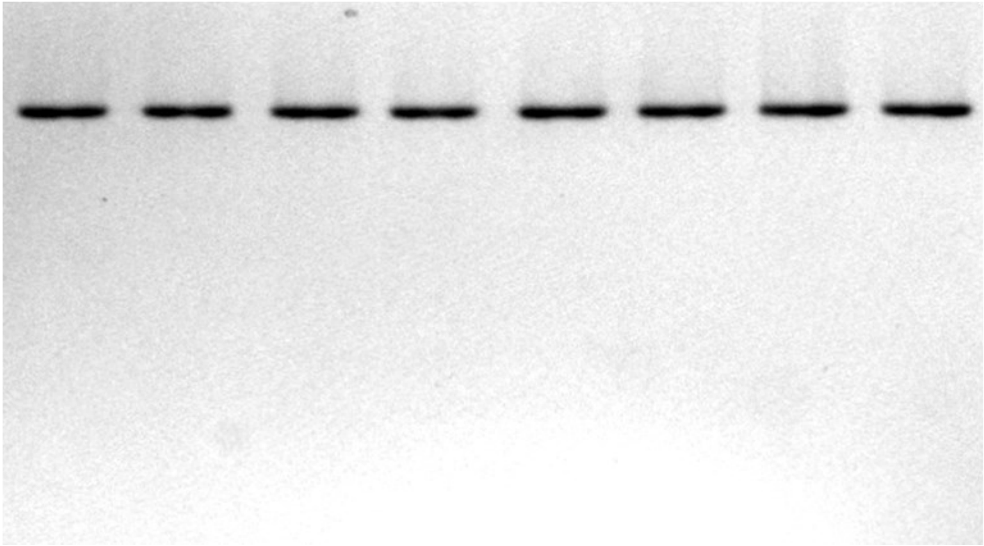

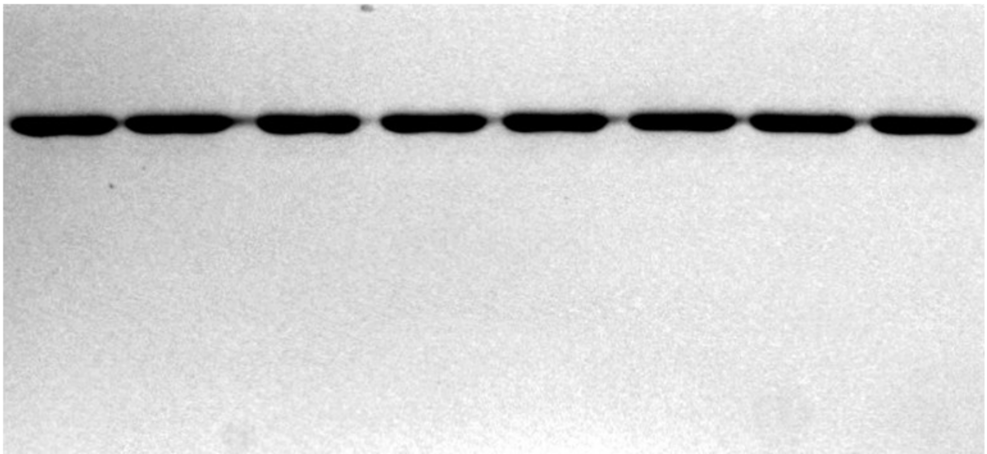

Figure S11. c-NF-κB

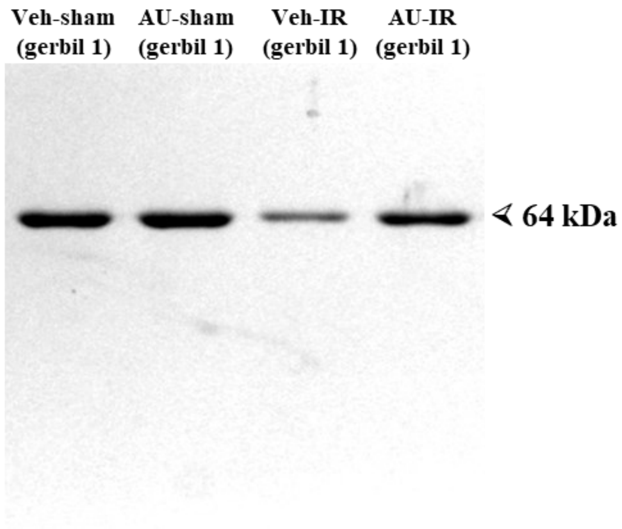

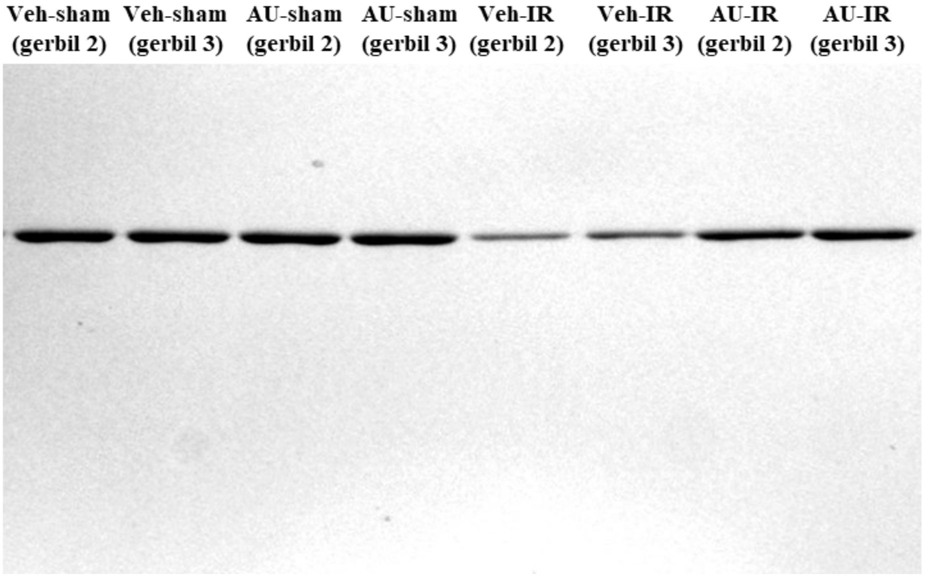

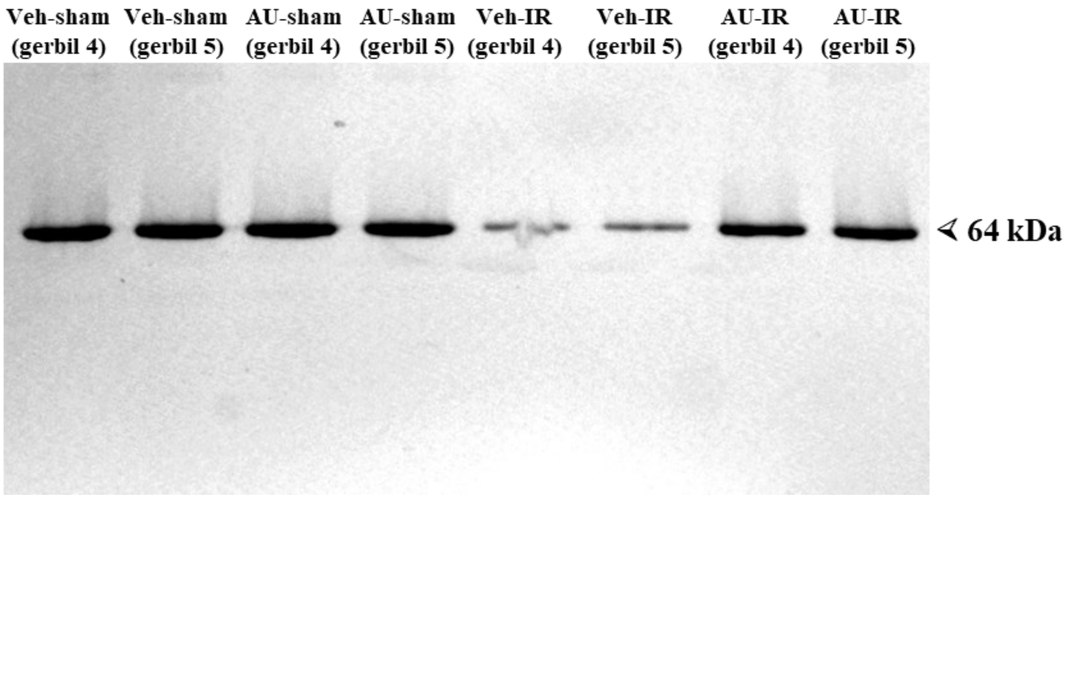

Figure S12. β-actin for c-NF-κB

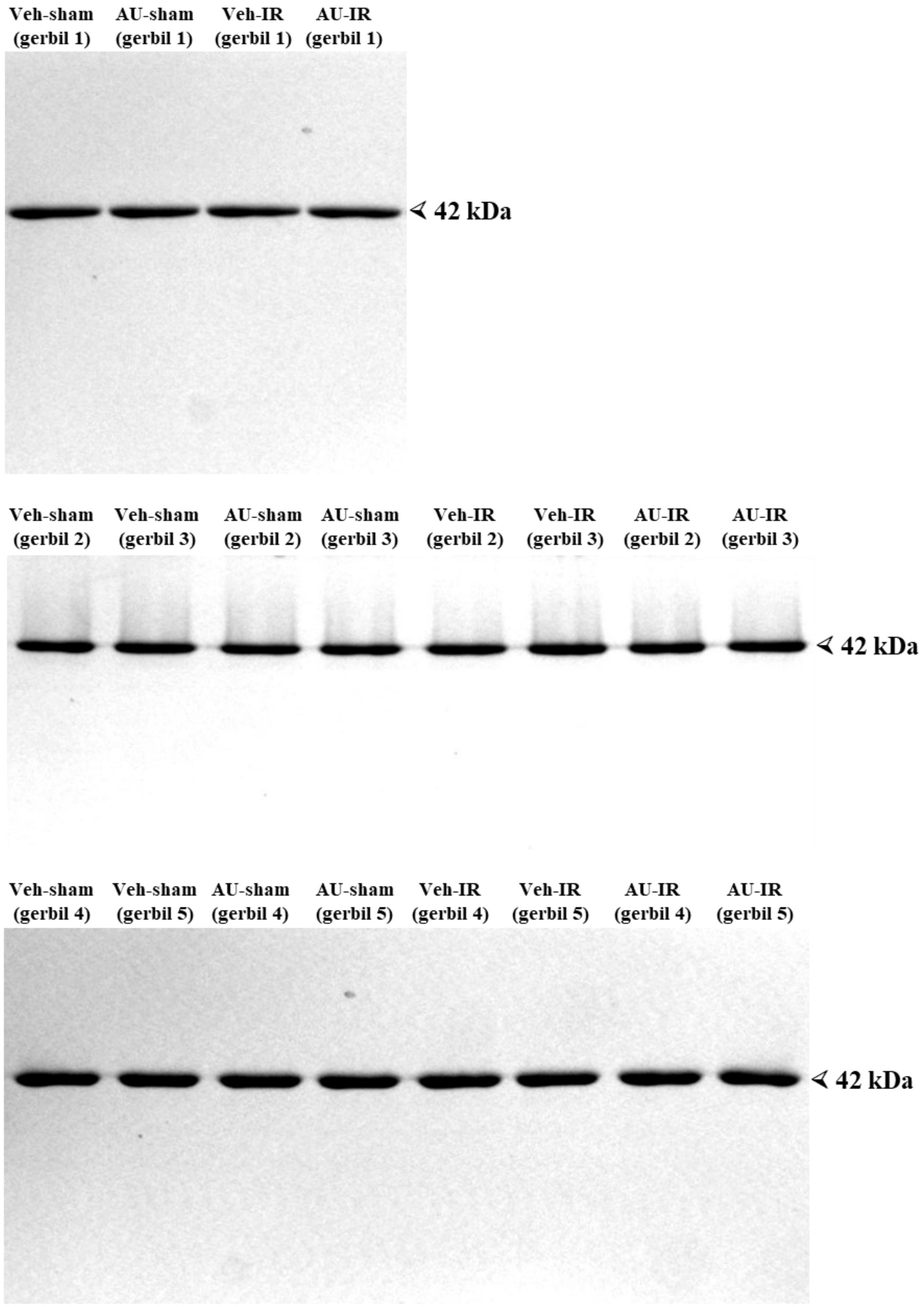

Supplementary Figures S5-12. Original images of Western blot data in Figure 4
